# Circulating tumor cells clusters and their role in Breast cancer metastasis; a review of literature

**DOI:** 10.1007/s12672-024-00949-7

**Published:** 2024-04-01

**Authors:** Zeinab S. Sayed, Mohamed G. Khattap, Mostafa A. Madkour, Noha S. Yasen, Hanan A. Elbary, Reem A. Elsayed, Dalia A. Abdelkawy, Al-Hassan Soliman Wadan, Islam Omar, Mohamed H. Nafady

**Affiliations:** 1 Faculty of Applied Medical Science, Misr University for Science and Technology, 26Th of July Corridor, 6Th of October, Giza Governorate, Postal Code: 77 Egypt; 2 Technology of Radiology and Medical Imaging Program, Faculty of Applied Health Sciences Technology, Galala University, Suez, 435611 Egypt; 3 Radiology and Imaging Technology Department, Faculty of Applied Health Science Technology, Delta University for Science and Technology, Gamasa, Al Mansurah, Egypt; 4 Faculty of Medicine, Benha University, Qalyubiyya, Egypt; 5 Faculty of Dentistry, Sinai University, Arish, North Sinai Egypt; 6 Faculty of Pharmacy, South Valley University, Qena, Egypt; 7 Radiation Sciences Department, Medical Research Institute, Alexandria University, Alexandria, Egypt; 8 Faculty of Applied Health Science Technology, Misr University for Science and Technology, 6th of october, Egypt

**Keywords:** Breast cancer, Metastasis, Circulating tumor cells, CTC clusters, CTC detection, CTC biology, CTC prognosis

## Abstract

Breast cancer is a significant and deadly threat to women globally. Moreover, Breast cancer metastasis is a complicated process involving multiple biological stages, which is considered a substantial cause of death, where cancer cells spread from the original tumor to other organs in the body—representing the primary mortality factor. Circulating tumor cells (CTCs) are cancer cells detached from the primary or metastatic tumor and enter the bloodstream, allowing them to establish new metastatic sites. CTCs can travel alone or in groups called CTC clusters. Studies have shown that CTC clusters have more potential for metastasis and a poorer prognosis than individual CTCs in breast cancer patients. However, our understanding of CTC clusters' formation, structure, function, and detection is still limited. This review summarizes the current knowledge of CTC clusters' biological properties, isolation, and prognostic significance in breast cancer. It also highlights the challenges and future directions for research and clinical application of CTC clusters.

## Introduction

**Fig. 1:**
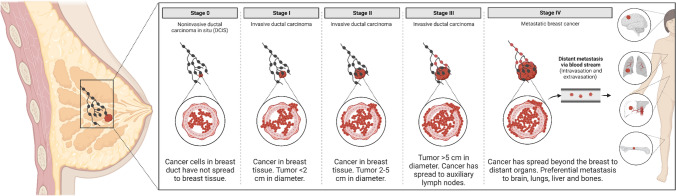
Stages of breast cancer. Stage (0) There is no spread to other breast tissues. Stage (I) cancer size in breast tissue is less than 2 cm. Stage (II) cancer size in breast tissue is 2–5 cm. Stage (III) tumor is more than 5 cm, and the cancer has spread to auxiliary lymph nodes. Stage (IV) Cancer has spread beyond the breast to distant organs. Preferential metastasis to the brain, lungs, liver, and bones

Breast cancer (BC) has been identified as one of the most widespread cancers among women worldwide [1–5]**.** BC can be classified into two primary categories: carcinomas and sarcomas [5]. Carcinomas originate from the epithelial component of the breast and contain terminal ducts and cells that line the lobules. At the same time, sarcomas are another group of breast cancers that arise from the breast's stromal components and consist of myofibroblasts and blood vessel cells [5, 6]. Some factors aid the development of BC, such as age, hormone status, genetic predisposition, and family history [7]. BC has some stages to develop [8]. In Stage 0, no significant change occurs; in Stage I, the tumor mass is considered minor and has no spread outside the breast. In stage II, the tumor is usually less than 2 cm in diameter, and it may exist in axillary lymph nodes; in stage III, the size of the tumor varies, meaning it can be any size, and at this stage, inflammation and change in skin color of the breast may occur due to the spread of the tumor to the chest wall. Stage IV is the most severe, as cancer metastasizes to distant areas like the lungs, bones, or brain, marking the start of metastatic breast cancer (MBC) as it invades far-reaching organs [8, 9] (Fig. 1).

BC metastasis is a complicated process involving multiple biological stages [10]. Initially, BC cells begin to separate from the extracellular matrix (ECM) [8]. They then start to invade and migrate locally [11]. Subsequently, a metastatic cascade is triggered when cancer cells separate from adjacent cells and the basement membrane [8, 12]. Cells that can invade the surrounding tissue employ proteolytic enzymes to degrade the ECM and facilitate invasion [13]. Cancer cells then attach to the endothelial wall and enter the circulation through lymph or blood vessels, eventually reaching other organs [8]. When some cancer cells break away from the original tumor or metastatic tumor, circulating tumor cells (CTCs) are formed from these separated cancer cells [14–16]. These cells can travel through the bloodstream, developing new metastases in other body parts [17]. CTCs were first discovered by an Australian physician, Thomas Ashworth, in 1869 [18].

Over the years, more has been learned about these rare cells in small numbers in the blood (a few per 10 ml) [19]. CTCs can circulate either as single cells or in clusters [20]. Those circulating in clusters appear to have developed mechanisms to survive the harsh bloodstream conditions [20–22]. Several studies have explored the composition of CTC clusters, revealing that they consist of two types of cells: tumor cells (homotypic) and non-tumor cells (heterotypic), such as mesenchymal cells, epithelial cells, pericytes, immune cells, platelets, and cancer-associated fibroblasts. These non-tumor components are critical in enhancing the clusters' survival rate and metastatic advantages [20, 23–32]. Isolating CTCs is relatively straightforward, as it can be done through a blood draw, making it more accessible than other methods like biopsy and imaging [33]**.**

Furthermore, several respected studies have established a correlation between the formation of CTC clusters and a poorer prognosis and lower patient survival rates [20, 21, 34–38]. Therefore, focusing on various aspects of CTCs, including understanding their biology, function, and detection and isolation methods, would be a valuable pursuit to gain deeper insights into BC and its metastasis. In this narrative review, we demonstrate the association between BC metastasis's potential incidence and the formation of CTC Clusters. We begin by offering insights into the biology, functionality, and mechanisms of CTC Clusters. This leads to exploring the potential utility of CTC clusters as predictive tools for monitoring therapeutic responses and forecasting patient prognoses in BC. Additionally, we provide general information about BC and its stages.

### Biology of CTC clusters

**Fig. 2:**
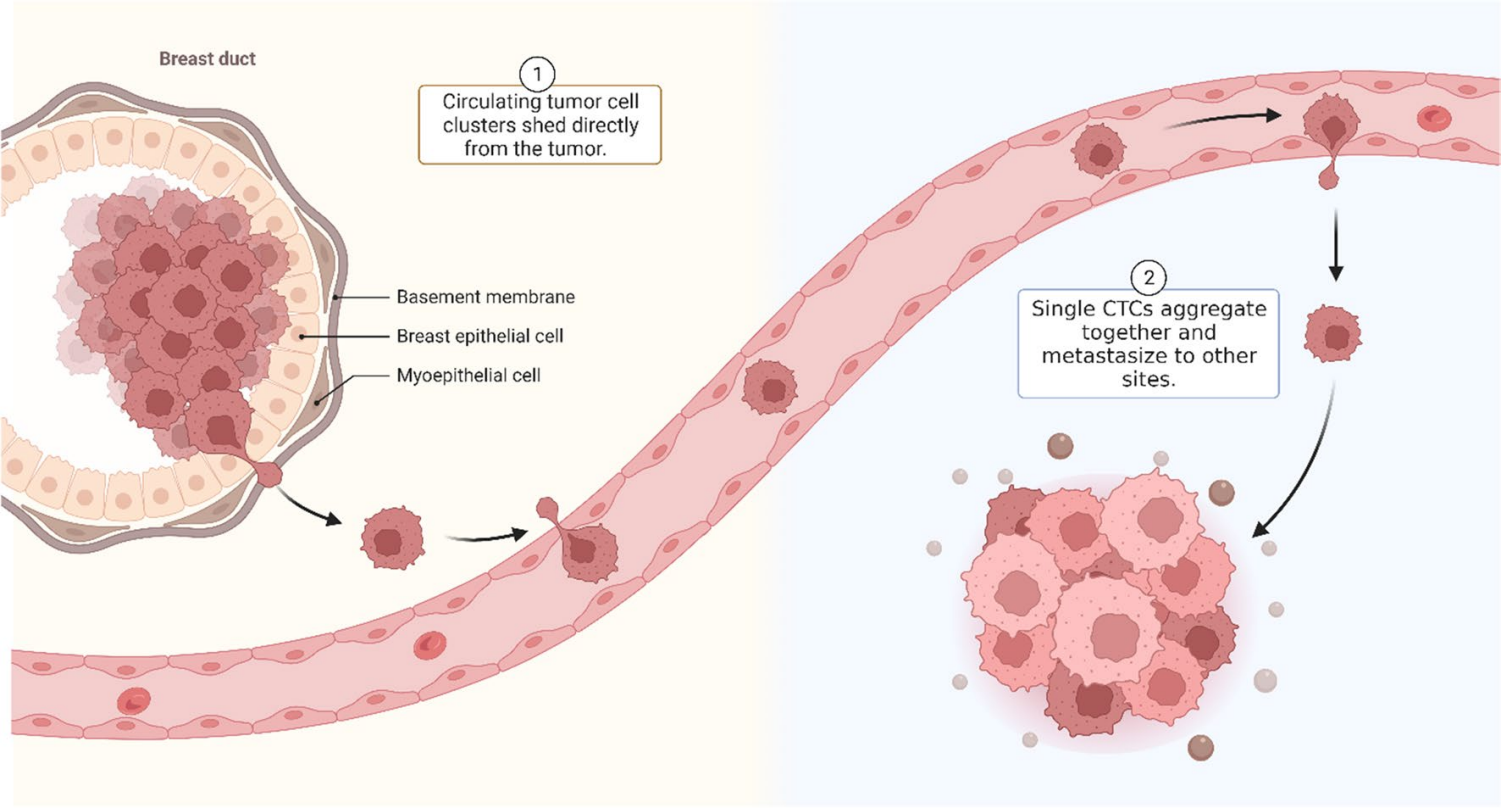
Caption CTC clusters metastasis. Circulating tumor cell (CTC) clusters are a group of tumor cells that move together in a cancer patient's bloodstream and have strong cell–cell contacts. These cells shed directly from the primary tumor "self-seeding" (1), or they can be formed when single CTCs in the circulation aggregate together and form clusters of tumor cells in other tissues (2)

CTC clusters are tumor cells that move together in a cancer patient's bloodstream and have strong cell–cell contacts [39, 40]. Pathologist Rudolf Virchow first postulated CTC clusters in 1858, suggesting that the arrest of tumor microemboli in the vasculature could be the cause of metastasis [41, 42]. In 1954, Watanabe demonstrated the role of CTC clusters in the progression of tumor metastases by injecting bronchogenic carcinoma cells into the jugular veins of mice. He found that CTCs developed metastases in clusters rather than as individual cells [43]. One of the most essential questions about CTC clusters is their origin. There are two main hypotheses; the first is that CTC clusters shed directly from the primary tumor (Self-seed), and the second is that they can be formed when a single CTC in the circulation aggregates together [23, 44] (Fig. 2). Cheung et al. tested the last hypothesis by engrafting equal mixtures of tandem dimer TD-Tomato and cyan blue fluorescent protein (CFP)-expressing breast tumor cells in the same mammary fat pad [45]. The frequent polyclonal metastatic seedling discovered by the authors was likely caused by oligoclonal CTC clusters [46]. Additionally, they found no evidence of bicolored metastasis in the lungs following intravenous injection of a single fluorescent cell or grafting fluorescent tumor cells into a mouse's distinct mammary fat pad [46]. It should be mentioned that primary and metastatic tumors can both emit CTC clusters, which can serve as local or distant "self-seeding" sources for malignancies [47].

Many molecules that help in tumor cell aggregation in BC form CTC clusters. It has been found that keratin 14 and plakoglobin, both linked to desmosomes and hemidesmosomes, are essential for CTC clusters formation [23]. Distal metastases and CTC clusters formation decreased by inhibiting these proteins [20, 45]. Also, BC cell aggregation is influenced by interactions between galectin-3 and Thomsen-Friedenreich glycoantigen [48]. Furthermore, several pro-inflammatory cytokines, like interleukin-6 and tumor necrosis factor-α, may enhance the clustering of tumor cells [49].

### CTC clusters categories

CTC clusters can be divided into two categories: homotypic and heterotypic CTC clusters. Tumor cells are the only component of homotypic clusters. s Heterotypic clusters are composed of an aggregation of cancer cells and non-cancerous cells like blood cells, endothelial cells, platelets, and fibroblasts [20, 21, 50, 51]. Homotypic CTC clusters represent a small percentage (1–30%) of all CTC events when detected in the peripheral circulation of patients or mice models, and their presence is dependent on the tumor size, disease stage, and molecular features [20, 51–53]. In BC, homotypic clustering of CTCs can be regulated by CD44 homophilic interactions, which activate various signaling pathways such as OCT4, EGFR, and the p21-activated kinase 2 /focal adhesion kinase (FAK) [22, 54]. Intercellular Adhesion Molecule 1 (ICAM1), a recently discovered stemness-promoting adhesion molecule, is also involved [54]. Other studies have proved that intratumor hypoxia can cause cluster development [55]. OCT4, SOX2, and NANOG are essential stemness genes enhanced by CTC homotypic clustering, encouraging stemness and clustered cell proliferation [56]. The development of CTC clusters may be enhanced by the cooperation of several adhesion and junction proteins [21].

It has been found that heterotypic CTC clusters are vital in seeding tumor clusters and maintaining resistance against host immune responses through their non-cancerous cells [57–60]. CTC-neutrophil clusters appear significant in the metastatic process; IL-1 b and IL6 are involved in a cytokine-receptor interaction that mediates this [60]. The intercellular connections that hold CTC-neutrophil clusters together depend on VCAM-1 [60]. In BC animal models, a decrease in neutrophils has been associated with a delay in releasing CTCs and CTC-neutrophil clusters from the primary tumor site. This reduction has also been linked to a delay in the development of metastases and a shorter overall survival rate in mice [44]. One intriguing observation is that the first contact between CTCs and neutrophils seems to occur at the primary tumor site, not in the bloodstream. Tumor-infiltrating neutrophils leave the primary cancer site and cancer cells and enter the bloodstream as CTC-neutrophils clusters [60]. Another type of heterotypic CTC cluster that has been found to promote the metastatic ability of CTC clusters is the CTC-platelets cluster. CTC clusters in the circulation are physically shielded by platelets from shear forces and immune responses [61, 62]. In many studies, the relationship between CTCs and platelets has also been established in patient samples by identifying the expression of platelet markers (such as SELP, ITGA2B, SPARC, and ITGB3) from total RNA extracts of CTCs (both single and clustered) [20, 60, 63]. Additionally, heterotypic CTC clusters in migration or circulation show the presence of cancer-associated fibroblasts, previously known to promote cancer invasion and spread from the primary tumor [64]. This was demonstrated in a mice experiment where the ability of the mice to develop lung metastases was decreased by removing fibroblasts from the clusters [24].

### Relationship between CTCs and microorganisms

Transitioning from the cellular dynamics of CTC clusters, we explore the pivotal role of microorganisms in influencing breast cancer metastasis. Microbial interactions with CTCs present a complex layer of regulation, impacting tumor progression and metastatic potential. This relationship underscores the e microbiome's influence on cancer pathways, including the critical process of epithelial-mesenchymal transition (EMT), which facilitates CTC migration and survival. By understanding these microbial interactions, we gain insights into novel therapeutic avenues targeting the microbiome to mitigate metastasis.

A growing field of research examines the nature of the microbiome in metastatic disease. The human commensal microbiota comprises every type of microbe that lives in the human body, including viruses, bacteria, protozoa, and fungi [65]. The growth of the immune system and the host's defense against several diseases, including cancer, are influenced by gut microbes [66]. Interaction between the immune system and the microbiota of tumor cells may boost the chance of cell survival and stimulate tumor cell migration [67]. The microbiota influences the EMT, a critical stage for CTC migration and survival. Toxins produced by bacteria cause EMT [68]. Certain microorganisms, such as Fusobacterium nucleatum, E. faecalis, and Bacteroides fragilis, can remove the transmembrane adhesion protein E-cadherin from epithelial cells, which promotes the growth of colonic epithelial cells [69]. It's currently unclear how certain microbiota compositions, the inflammatory response, and treatment resistance connected to EMT are related [70]. Changes in the gut microbiota could boost EMT via the WNT, TGFβ, and Notch signaling pathways and SNAIL, Slug, ZEB1, Twist, and ZEB2 transcription factors, resulting in invasive and metastatic cancer processes [71–73].

The generation of inflammatory cytokines and macrophage activation were facilitated by antibiotic-induced gut dysbiosis, which in turn promoted EMTin colorectal cancer [74]. Bacteria recognized to be implicated in the advancement of colorectal cancer include Salmonella enterica, Bacteroides fragilis, Fusobacterium nucleatum, and Enterococcus faecalis. These bacteria produce virulence factors that aid in the growth of cancer and EMT[75]. In the case of BC, the well-known role that estrogens play in the development of hormone-dependent BC is impacted by the gut microbiota's influence on estrogen metabolism [76]. Bacteria in the phylum Proteobacteria, including Escherichia and Shigella, express b-glucuronidase enzymes that facilitate sexual hormone reabsorption through the enterohepatic pathway. This process raises circulating estrogen levels, affecting BC growth [77, 78]. A study of fecal samples revealed that the gut microbiome of BC patients with bone metastases had a higher level of Acinetobacter, Bacilli, Collinsella, Epsilonproteobacteria, Campylobacter, Lactobacillales, Streptococcus, Veillonella, Pseudomonadales, and Moraxellaceae than control samples. On the other hand, the fecal sample analysis from individuals with metastatic disease revealed much-reduced concentrations of Paraprevotella, Clostridia, Megamonas, Akkermansia, and Gemmiger. As a result, researchers hypothesized that a correlation between the occurrence of bone metastases and reduced levels of Megamonas and Akkermansia may exist [79].

Since advancements in genome sequencing in recent years, researchers have shown that microbiomes, sometimes known as tumor microbiomes, are present in solid human tumors [80–82]. Compared to other body regions, the bacterial pattern seen in normal BC is distinct and includes Actinobacteria, Firmicutes, Proteobacteria, and Bacteroidetes [83–85]. BC formation and progression are probably influenced by the different microbiota found in breast tumors compared to normal counterparts [76]. According to Fu, Aikun, et al., the intracellular microbiota present in tumors plays a role in the reorganization of the actin cytoskeleton of tumor cells, which increases the cells' resistance to fluid shear stress upon entry into the systemic circulation. This ultimately leads to an increased risk of metastatic colonization and cancer aggressiveness [86]. The intratumoral microbiome can potentially contribute to BC recurrence and metastasis by promoting tumor stem cell activity, transforming epithelial cells into mesenchymal cells, and facilitating cell migration [87]. According to research by Parhi and colleagues, Fusobacterium nucleatum colonizes BC through Gal-GalNAc, which is present in large amounts in tumor cells. It then encourages the growth and spread of BC by preventing T-cell aggregation in the tumor's surrounding tissue. [88]. In the murine spontaneous breast tumor model, the decrease of the tumor microbiome brought on by antibiotic therapy prevented the formation of lung metastases. It was shown that the intratumoral delivery of certain bacteria, including Ligilactobacillus animalis, Spilopsyllus cuniculi, and Staphylococcus xylosus, raised the number of lung metastases in mice without changing the main tumors [86].

### CTC clusters and metastasis

Even though metastasis is the primary cause of mortality among BC patients, research is still ongoing to understand the mechanisms that promote the spread of tumor cells and the formation of metastases. However, this may be assisted by studying the mechanism of action of CTCs, the progenitors of metastatic spread. As known, more than 90 percent of malignant cells shed into blood circulation have a low probability of surviving; only a tiny fraction of individual CTC or clusters can survive [44]. A study by Liu et al. has demonstrated that CTC clusters are more potent in facilitating metastasis formation than single CTCs, particularly in triple-negative patient-derived BC models (PDXs) [33]. Strong cell–cell connections enable these tricky cells to disseminate in clusters, avoid anoikis, a form of apoptosis, and supply survival factors that encourage their metastatic potential [39, 57]. In BC, the proteins circulating galectin-3 and CD44-mediated signaling pathways, as well as cancer-associated mucin1 (MUC1), interact altogether, inhibiting anoikis of clusters in the circulation, promoting their aggregation and enhancing their seeding to distant organs [22, 89]. CTCs initiate their extravasation process by slowing down within tiny capillaries, clinging to the endothelial lining of vascular structures, and traversing the endothelium [90]. Two primary mechanisms have been proposed to explain the extravasation of circulating tumor cells CTCs. The first mechanism is a physical blockage of the CTCs in smaller diameter capillaries due to of the expression of ligands and receptors on endothelial cells and CTCs. The second mechanism involves cell adhesion to the endothelium in veins larger in diameter [91].

In addition, tumor cells can create a "premetastatic niche" [92] via which systemic signals (cytokines, exosomes, and extracellular matrix remodeling enzymes) are released from the primary tumor, providing a more hospitable microenvironment for CTCs [93]. Consequently, this mechanism is considered necessary for their work, improving their adaptability to various microenvironments and helping CTC clusters metastasize easily. Therefore, it has been proved that the poor prognosis and the increasing metastatic phenotype observed in BC patients are linked to the epigenetic signature identified in clusters of CTCs; these clusters are characterized by hypomethylated regions that are abundant in binding sites for embryonic stem cell transcription factors [56].

### CTC clusters immunology

Immunologically, CTC clusters possess the unique ability to aggregate with different immune cells (heterotypic clusters), which act as a physical shield, providing a protective barrier against immunological surveillance. Interestingly, it has been discovered that neutrophils, a subtype of white blood cell, directly interact with breast CTCs, influencing the transcriptional profile of tumor cells, promoting the progression of the cell cycle in the blood, and hastening the seeding of metastases. Studies have indicated that individuals diagnosed with BC who presented with a minimum of one CTC cluster containing neutrophils had a significantly poorer prognosis than those with less than five CTCs in a volume of 7.5 ml of peripheral blood [60]. Indeed, heterotypic clusters in BC can metastasize quickly due to the presence of stroma-derived cells and platelets [94]; the latter coats clusters as a physical shield to protect them from shear forces in the circulation, collisions with other blood cells, and immunological reactions mediated by cytotoxic natural killer (NK) and T cells [61]. Additionally, this aggregation can facilitate CTC migration through the endothelial barrier by improving their adherence to the vasculature [95].

Moreover, platelets can maintain the integrity of CTC clusters using paracrine secretion of substances such as transforming growth factor (TGF), a well-established inducer of theEMT process. This process is fundamental to developing intrinsic heterogeneity in CTCs [96]. Predominantly, Cancer cells initially colonize through a reversible process known as a mesenchymal-to-epithelial transition (MET), which facilitates their colonization in metastatic foci [97]. The CTC cluster exhibits both epithelial and mesenchymal characteristics simultaneously in MBC patients [98, 99]; the hybrid epithelial-mesenchymal phenotype that is observed endows these aggregates with a considerable degree of plasticity, thereby conferring a trait of advantageous survival, as has been proposed in the literature [100]. The underlying mechanism appears to be the combination of mesenchymal traits that favor a migratory phenotype and the maintenance of cell–cell junctions in epithelial cells [94].

### Comparison between single CTCs and CTC clusters in BC metastasis

A collection of cancerous cells, consisting of more than two or three (Even up to a hundred, which exhibit significant cell–cell interactions), have been identified as a CTC cluster inside a cancer patient's bloodstream. Although extremely metastatic, it seems to be exceedingly uncommon; in a mouse model, clusters were demonstrated to make up around 50% of BC metastases despite making up just 2–5% of total CTCs. Moreover, the metastatic potential of these clusters has been estimated to be between 23 and 50 times more than that of single CTCs [44]. Additionally, unlike single CTCs in many cancer classifications, the occurrence of CTC clusters and the size of their clusters are linked to worse clinical outcomes [20, 36, 37, 101]. It was also proposed that maintaining robust cell–cell adhesions might shield cell clusters from anoikis (apoptotic cell death), brought on by a relative lack of adherence [102]. Therefore, CTC clusters could have benefits in terms of survival both during circulation and during dispersion. Studies have demonstrated that CTC clusters have less apoptosis, increased survival, and colony-forming capability [20, 45]. Conversely, because of their interception via small vessels, the duration of CTC clusters in circulation is incredibly brief (much briefer than individual CTCs, which may persist in circulation for only a few hours [103]. In vivo, flow cytometry determined the clearance rate of the tagged clusters and individual CTCs from the blood [20].

Further research shows that CTC clusters differ from single CTCs in gene profile expression and dispersion methods. Cell–cell junctions may be crucial in constructing and preserving CTC clusters in the circulatory system, according to transcriptome investigations, which have revealed that these structures retain epithelial characteristics. Plakoglobin and keratin 14 (K14), two proteins participating at desmosome and hemidesmosome junctions, have shown higher expression in clusters than in single cells [20, 45]. While single-cell whole-genome bisulfite sequencing (sc-WGBS) investigation of DNA methylation patterns across both individual and clustered CTCs has demonstrated that clustering causes hypomethylation of Linking locations associated with stemness as well as prevalence regulatory, which include SOX2, SIN3A, OCT4, and NANOG. Moreover, hypermethylation of Polycomb target genes is also shown to increase stemness and proliferation concurrently [56]. The CTC cluster's impact on MBC survivability has also been noticed by Jansson et al.[36], who demonstrated that follow-up samples of patients having CTCs and CTC clusters following systemic irradiation had the worst prognosis in terms of progression-free survival (PFS) and overall survival (OS) compared to those without such cells. In the Mu et al. study [104], patients with BC phases III and IV had a lower PFS when their baseline numbers of both single CTCs and CTC clusters (classified as C2 CTCs) were high. According to Paoletti et al.'s research, CTC clusters are significant in metastatic triple-negative BC (TNBC) and apoptosis [105] Table 1.

**Table 1:** Studies comparing the biological and pathological characteristics of CTCs and CTC clusters

Study	Tumor type	Metastatic potential	CTCs and CTC Cluster detection method	Study main findings	Reference
Cheung et al. (2016)	Breast cancer mouse model	The ex vivo colony formation increased by > 15-fold, and in vivo metastasis development increased by > 100-fold when tumor cells were aggregated into clusters	Multicolor lineage tracing	In a mouse model, multicolored tumor cell clusters were observed across all main phases of metastasis, including collective invasion, local dissemination, intravascular emboli, circulating tumor cell clusters, and micrometastases	[45]
Donato et al. (2020)	Breast cancer mouse model	The average number of cells in hypoxic CTC clusters was larger than that of normoxic CTC clusters, with 5.3 cells per hypoxic CTC cluster and 2.82 cells per normoxic CTC cluster	Live imaging of HIF1a reporter	In a mouse model, Most CTC clusters are hypoxic; conversely, most single CTCs are normoxic	[55]
Szczerba et al. (2019)	Patients with breast cancer and mouse models	Among breast CTCs, CTC-neutrophil clusters are the most effective subpopulation for metastasis formation, and a patient's bloodstream containing these cells is linked to a poor prognosis	Parsortix microfluidic device	- In breast cancer patients, those with 7.5 ml of peripheral blood containing at least one CTC-neutrophil cluster had a substantially worse progression-free survival than those with five or more CTCs in 7.5 ml of peripheral blood - Research revealed that mice administered CTCs from CTC–neutrophil clusters experienced a significantly lower survival period and overt metastasis than those injected with CTCs alone	[60]
Aceto et al. (2014)	Breast cancer mouse model	CTC clusters have been estimated to be between 23 and 50 times more than that of single CTCs	Herringbone HB CTC-Chip	In a mouse model, clusters were demonstrated to make up around 50% of breast cancer metastases despite making up just 2–5% of total CTCs. Still, CTC clusters have been estimated to be between 23 and 50 times more than that of single CTCs and contribute to approximately half of all metastatic lesions in orthotopic breast cancer models	[20]
Duda et al. (2010)	Mouse lung cancer cell line	The presence of fibroblasts in clusters makes cancer cells more viable in the bloodstream and at the secondary location	Whole-mount fluorescence microscopy	Using diphtheria toxin treatment 24 h after cell infusion to eliminate carcinoma-associated fibroblasts (CAFs), the number of metastases assessed two weeks after infusion did not change significantly. These findings demonstrate the ability of tumor-associated fibroblasts to stimulate lung metastasis. This advancement cannot occur when the CAFs are not directly associated with the cancer cells within the metastatic foci	[24]
Liu et al. (2019)	Patients with breast cancer and mouse models	CTC clusters are more potent in facilitating metastasis formation than single CTCs, particularly in triple-negative patient-derived BC models (PDXs)	Cell search and fluorescence microscopy	-CTC clusters are more potent in facilitating metastasis formation than single CTCs, particularly in triple-negative patient-derived BC models (PDXs) -The breast cancer stem cell marker CD44 was highly expressed by aggregating tumor cells, encouraging carcinogenesis and polyclonal metastases - The interactions mediate tumour cluster aggregation between CD44 homophiles and, subsequently, CD44–PAK2. That will encourage the creation of new targeted techniques to prevent polyclonal metastasis and result in novel biomarker applications that predict prognosis	[22]
Murlidhar et al. (2017)	Patients with surgically resectable (clinical stage I-III) lung cancer	The cells within CTC clusters can avoid cell death, given their prognostic relevance and capacity for metastasis. CTC clusters have been demonstrated to be linked to a poor prognosis	Microfluidic device	In early-stage lung cancer, (CTCs) can help predict an early relapse and assist in the early diagnosis of metastases. A notably greater quantity of CTCs in the (PV) blood has been discovered than in the Preoperative (Pe) blood. Gene ontology analysis enriched cell migration and immune-related pathways in CTC clusters, indicating a possible survival benefit of the clusters while in circulation	[106]
Gkountela et al. (2019)	BC patients and mouse models	CTCs' ability to shape clusters has been connected to expanded metastatic potential	Whole-genome bisulfite sequencing (sc-WGBS) investigation	Demonstrated that clustering causes hypomethylation of Linking locations associated with stemness and prevalence regulatory, including SOX2, SIN3A, OCT4, and NANOG. Moreover, hypermethylation of Polycomb target genes is also shown to increase stemness and proliferation concurrently	[56]
Paoletti et al. (2015)	Metastatic triple-negative breast cancer (TNBC)	Metastatic	CellSearch®	In correlational studies, CTC clusters are significant in metastatic triple-negative breast cancer (TNBC) and apoptosis	[107]
Mu et al. (2015)	Metastatic Breast cancer (Stage III-IV)	Metastatic	CellSearch®	Patients with breast cancer phases III and IV had a lower PFS when their baseline numbers of both single CTCs and CTC clusters (classified as C2 CTCs) were high	[35]
Jansson et al. (2016)	Metastatic Breast cancer (Stage III-IV)	Metastatic	CellSearch®	In an observational study, follow-up (FU) samples of patients having CTCs and CTC clusters following systemic irradiation had the worst prognosis in terms of PFS and OS compared to those without such cells	[36]

## Methods for CTC cluster detection and isolation

A single CTC is uncommon in peripheral blood, and CTC clusters—about 3% of all CTCs are much less common [108]. Most CTC enrichment methods use specific markers to distinguish CTCs from leukocytes. The most prevalent epithelial cell markers are cytokeratins (CKs) and EpCAM (epithelial adhesion molecule) [109]. A hybrid epithelial-mesenchymal feature can be seen in CTC clusters, so using strategies based on epithelial markers is not helpful to CTC clusters [109]. ffinity-based and label-free approaches are to isolate CTCs and CTC clusters [110]. Affinity-based techniques for capturing CTC clusters employ cell surface markers and antigen–antibody [109]. The most frequently used biological approach for isolating CTCs or reducing leukocytes involves using antigen-specific antibodies attached to paramagnetic particles, selectively concentrating the target cells [109].

In contrast, label-free methods exploit the size differences between blood cells and CTC clusters [110]. Physical methods, as label-free methods, primarily use size, density, and electrical charge differences to distinguish CTCs from normal cells. Both approaches have benefits and drawbacks. For example, affinity-based methods can capture physically heterogeneous populations of CTCs due to their high purity. In contrast, label-free methods can capture biologically heterogeneous populations of CTCs due to their high throughput [110].

**Fig. 3:**
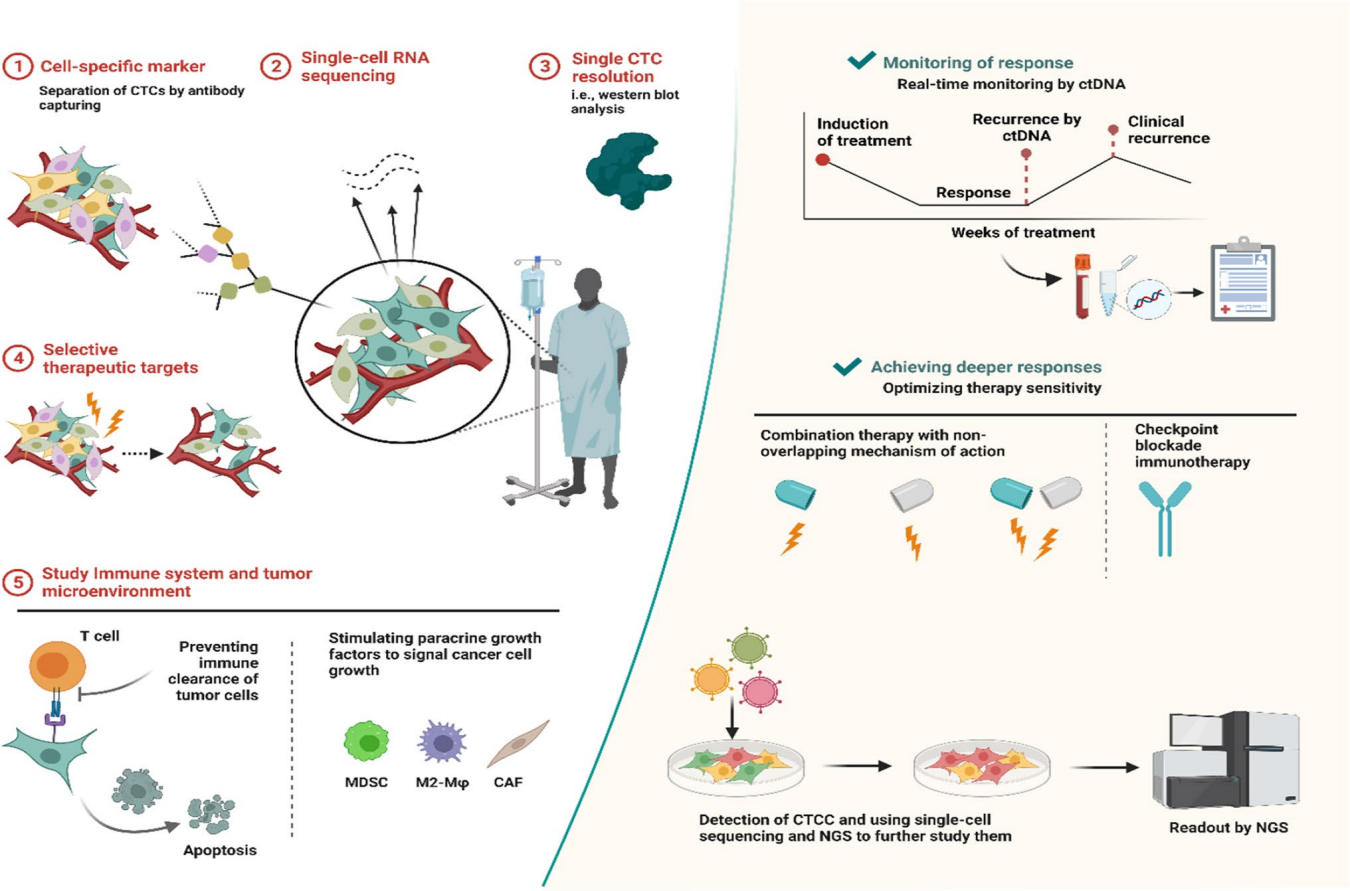
Basic techniques used for CTCs and CTC cluster detection

Generally, the advantages of the label-free method as the ISET: (i) It doesn't require antibody binding to maintain the CTC clusters' native state; (ii) it allows direct filtration of peripheral blood without preprocessing; (iii) It is capable of preserving CTC cluster integrity; (iv) It is less expensive than Affinity-based methods [23] f. The limitations of affinity-based methods are thatcapture requires optimal velocity and shear conditions forantibody–antigen binding [111, 112]. A very high shear may disrupt any bonds if formed, while a very low shear is conducive to non-specific cell binding and the limited expression of target antigens [23] (Fig. 3).

### Label-free methods

The ISET (Isolation by Size of Epithelial Tumor Cells), a label-free-method, is a widely used physical technique that captures most epithelial cells (20–30 µm) while allowing smaller leukocytes to pass through pores of a specific size and shape. Although this method is appropriate for detecting CTC clusters, which vary in size, it also retains larger leukocytes, reducing its specificity [109]. CTC clusters and CTCs are more prominent than leukocytes in the blood. Thus, their isolation by size is an easy process [113, 114]. This technique seemed not specific for isolating CTC clusters and stems from the filter's ability to retain larger leukocytes [109]. Generally, ISET technology can isolate CTCs from all types of cancers as whole cells without needing prior selection based on the immune system [115]. ISET is considered a more precise technique than other in vitro methods. In a study, 43% of patients with non-small cell lung cancer (NSCLC) were found to have CTC clusters through the use of ISET. However, the detection of CTCs was not fully achieved by Cell Search. Another study that utilized a similar ISET platform isolated CTC clusters from all lung cancer patients [116]. One study found that the ISET platform could detect clusters of CTCs in 2 out of 23 individuals with primary liver cancer [117]. Via utilizing ScreenCell® (ScreenCell, Sarcelles, France), an easy, non-invasive technology, to separate CTCs and CTC clusters by size on a microporous membrane filter, allowing for later characterization and sorting. The ScreenCell's filtration membrane devices can enable nucleated cells to pass while holding up CTCs and preserving the CTCs' morphology and structures [115].

**Fig. 4:**
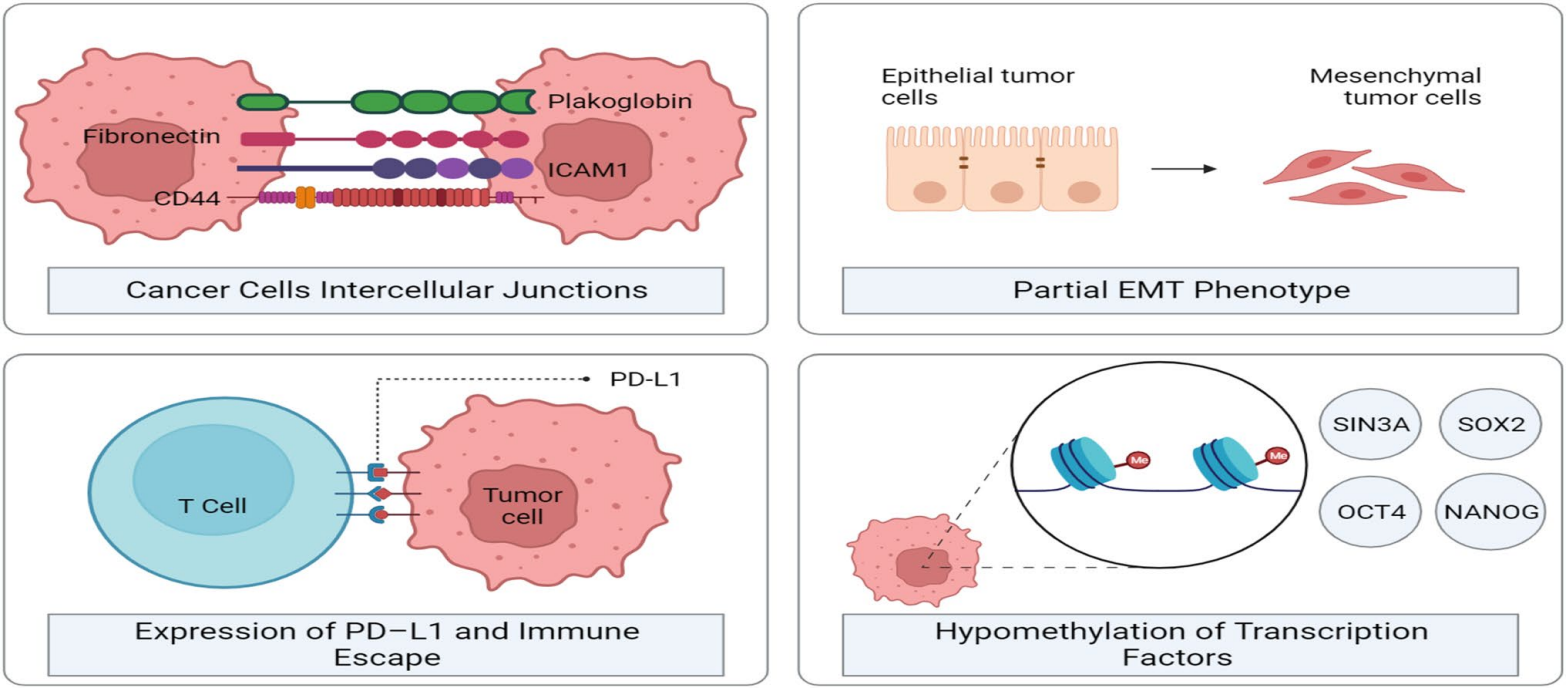
Caption Features of Homotypic Circulating Tumor Cell (CTC) Clusters. Once CTCs assemble into clusters, they establish favorable conditions for their survival through various mechanisms. Cancer cells preserve crucial intercellular junction molecules, including CD44, ICAM1, and plakoglobin, among others, to resist anoikis. The amalgamation of cancer cells induces hypomethylation in transcription factor binding sites associated with stem cells and proliferation, such as OCT4, SOX2, NANOG, and SIN3A. CTC clusters maintain a hypoxic environment, and there is a notable increase in the expression of PD–L1. Through these means, they successfully evade immune surveillance and lead to immune escape. Additionally, CTC clusters exhibit a mixed epithelial/mesenchymal phenotype, and when encountering small-diameter vessels, they organize into chains. Collectively, these characteristics contribute to the heightened metastatic capabilities of CTC clusters

The ScreenCell® device's circular filter is polycarbonate and has a smooth, flat, and hydrophilic surface. They sought to alter the pores' size, bringing it up to 15 m, to make it possible to filter huge CTC clusters selectively [118]. These devices are intended to isolate (a) fixed cells for cytological studies (ScreenCell® Cyto), (b) live cells for culture (ScreenCell® CC), and (c) molecular biology (ScreenCell® MB) [119]. Using a blood-filtration technique, we identified CTC clusters in 6 6 EBC patient samples and determined if DNA aberrations were present in 96% of the 48 examined clusters [120]. A unique flexible microspring array (FMSA) technology was recently developed to enrich viable CTCs according to their size, independent of antigen expression [121]. In colon, lung, and BC, FMSA detected CTC clusters of 2–20 tumor cells [121]. (Fig. 4).

### Affinity-based methods

The fundamental idea of technologies like CellSearch [122] involves the affinity between an antigen and an antibody. Antigens present on the membrane of CTC clusters are targeted by specific antibodies that can be immobilized onto a solid surface [123]. The optimal conditions for affinity-binding allow the antigens to attach themselves to the target antibodies. Then, depending on how they were captured, the bound cells can be separated for additional testing [124].

The identification of CTCs and CTC clusters is achieved through immunomagnetic techniques, wherein CTCs are marked with antigen-specific antibodies conjugated to magnetic beads [125–128]. CTCs are captured using antibodies that target specific markers on epithelial cells, such as CKs, EpCAM-specific antigens, tumor antigens, carcinoembryonic antigen (CEA), and human epidermal growth factor HER2 [109]. Cells bound by antibodies attached to magnetic beads can be separated from leukocytes using a magnetic field. These isolated CTCs can then undergo further analysis. Some systems used for this technology include EasySep from Stem Cell Technologies in Canada, Dynal Magnetic Beads from Invitrogen in the USA, and MACS (Magnetic Activated Cell Sorting) from Miltenyi Biotec GmbH in Germany [129–131]. The CellSearch System, authorized by the US Food and Drug Administration (FDA), is the only test available for clinically detecting CTCs. The AdnaTest, developed by AdnaGen AG in Germany, is used to isolate CTCs using a magnet and then lyse them to measure the expression of markers for MUC1, HER2, and GA73.3 surface glycoprotein-2, allowing for the identification of CTCs [132]. According to a research study, the use of CK and prostate-specific antigens as combined biomarkers for prostate cancer showed that the occurrence of CTC clusters was up to 80% [126]. Another study on colorectal cancer found that using immunomagnetic labeled CK antibodies, CTC clusters could be isolated from the peripheral blood of 68.8% of patients [127]. The antibody-based techniques are ineffective for CTC clusters because they have lower surface-area-to-volume ratios and the restricted expression of target antigens, decreasing antibody capture effectiveness [112].

**Fig. 5:**
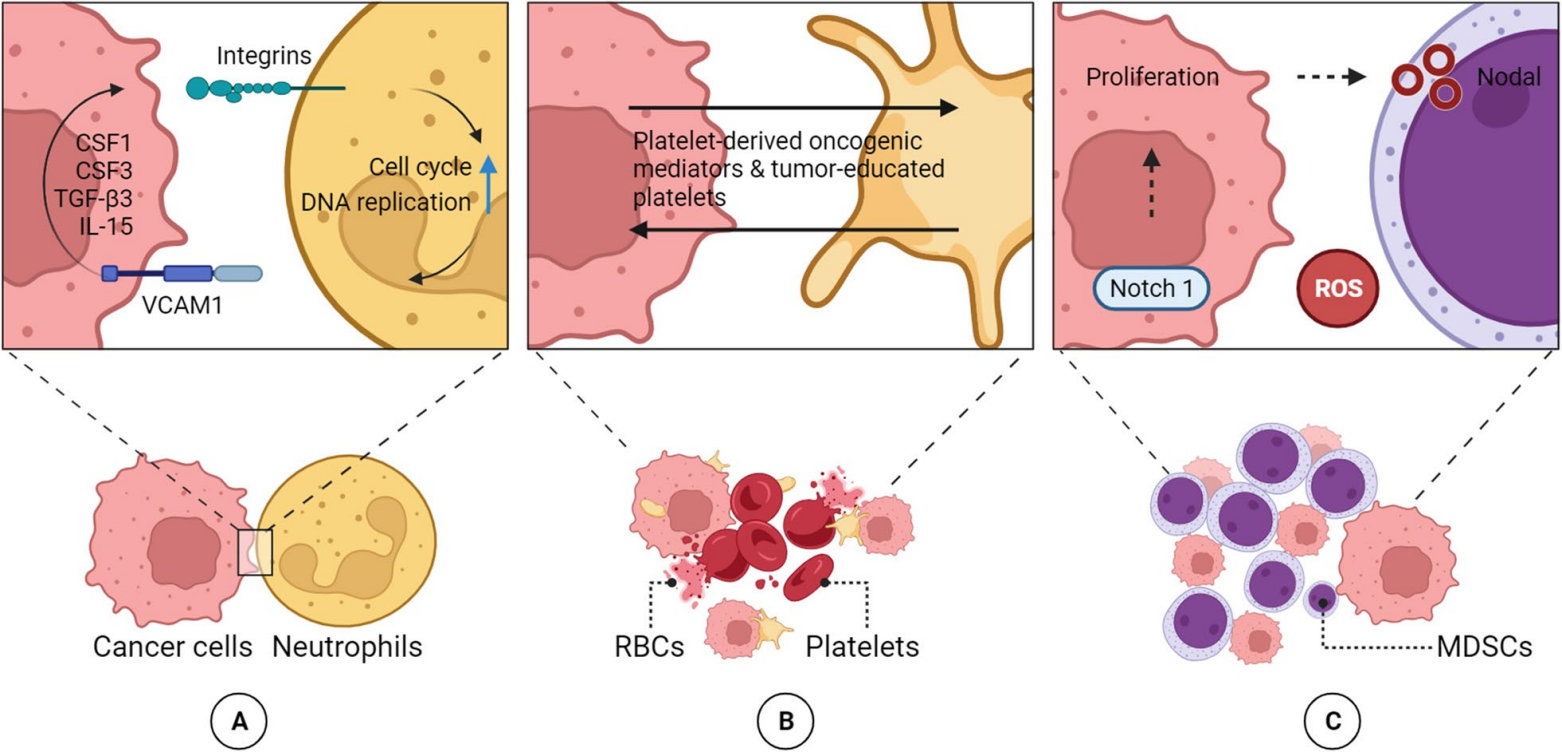
Caption Heterotypic Circulating Tumor Cell (CTC) Clusters. Formation of circulating tumor cell (CTC) clusters with diverse cell types. Heterotypic interactions occur between CTCs and white blood cells, such as neutrophils (**A**), platelets (**B**), and myeloid-derived suppressor cells (MDSCs) (**C**). These interactions play a role in facilitating immune evasion and promoting proliferation. Abbreviations: CSF1, colony-stimulating factor 1; CSF3, colony-stimulating factor 3; Il, interleukin; ROS, reactive oxygen species; TGF-β3, transforming growth factor-β3; VCAM-1, vascular cell adhesion molecule 1

In immunology, there are two types of selection: positive and negative. Positive selection involves using specific markers found on the surface of epithelial cancer cells. These markers are targeted by antibodies to identify CTCs in samples. Systems such as CellSearch® and Adnatest® use this approach to detect CTCs in breast carcinoma patients and monitor their response to chemotherapy or surgery [115]. At the same time, negative selection is a process that can be used to remove white blood cells and other types of blood cells. In the context of CTC enrichment, negative selection involves depleting white blood cells using antibodies that target specific biomarkers, such as CD66b or CD45 [115]. (Fig. 5.)

### Predictive value of CTC cluster counts in monitoring therapy response

#### CTC clusters in primary BC

CTCs,specifically CTC clusters, are considered potential metastatic seeds. The molecular characteristics of these cells, their persistence in circulation throughout therapy, and other prognostically significant information may be revealed by a greater understanding of these cells. While CTC clusters have been extensively studied in MBC patients [36–38, 133, 134], their properties in individuals with earlier stages of the disease remain less understood. Identifying early BC patients with CTC clusters may allow us to identify high-risk individuals who develop metastasis and expose them to cluster-targeting agents [135].

Krol et al. [136] have recently validated the existence of CTC clusters in the peripheral circulation of early BC patients who have not yet metastasized, and they are over three times more prevalent than in MBC patients [137, 138]. This investigation employed non-disruptive CTC visualization technology, identifying clusters in various subtypes: luminal-A-like, luminal-B-like, and HER2-positive, but not in triple-negative cases. The size of the detected CTC clusters varied, ranging from pairs to aggregates exceeding 50 cells. This finding emphasizes the early involvement of CTC clusters in BC and highlights the need for further research, particularly in developing early-stage, cluster-specific therapies. According to [139], marker-independent filtration technologies have revealed that 70% of early BC cases display CTC clusters, compared to just 20% of MBC patients. This suggests a higher incidence of CTC clusters in the early stages of BC compared to MBC, challenging the prevailing view that CTC clusters are mainly characteristic of advanced stages of the disease.

Additionally, CTC clusters were shown to be substantially more common in women with HER2-negative tumors [137, 139]. A related study involving six early-stage BC patients supports the findings of Krol et al. and Reduzzi et al. on the early appearance of CTC clusters in BC [120]. This research focuses on CTC clusters in early-stage patients, revealing notable tumor fractions and genomic differences compared to primary tumors. It emphasizes the significance of CTC clusters in early-stage BC, suggesting they may arise from varied tumor regions or micrometastases, and highlights the necessity for more concentrated research on cluster-specific therapies in the early stages of the disease.

In a meta-analysis involving 6825 BC patients from 49 studies, CTCs have been shown to have predictive power in metastatic patients and individuals with early-stage malignancies [140]. The data demonstrated that early-stage patients with CTCs face a higher risk of recurrence, as indicated by the pooled Hazard Ratio and a 95% confidence interval. Moreover, CTCs consistently serve as a reliable prognostic marker throughout treatment, unaffected by systemic therapy. According to Mu et al. [35], CTC clusters may have more predictive value (PFS) than CTC enumeration alone at baseline in patients with stages III and IV BC. Interestingly, it has been found that plakoglobin expression (a protein that constitutes both adherents' junctions and desmosomes) was higher in CTC clusters than in single CTCs and that its expression in primary tumors was associated with a significant reduction in distant metastasis-free survival (DMFS) [20]. In a similar study on 121 early-stage BC patients, plakoglobin expression in primary tumors has been identified as a significant prognostic indicator for distant metastasis in BC contexts,[141]. The findings from this study suggest that individuals exhibiting elevated levels of plakoglobin expression were associated with markedly poorer DMFS and exhibited a considerable increase in E-cadherin expression, a recognized marker of EMT [141]. These observations imply that plakoglobin not only correlates with the aggressiveness of breast cancer but also may be a more effective prognostic factor for predicting distant metastasis, highlighting its importance in evaluating patient outcomes. In light of the findings, exploring CTC clusters in primary BC highlights their potential as early indicators of metastasis risk, underscoring the necessity for further research. Such research should focus on elucidating the precise role of these clusters in the early stages of the disease and exploring their prognostic significance across different BC subtypes. This endeavor is crucial for identifying individuals at a higher risk of progression and integrating targeted therapeutic strategies.

#### CTC clusters in MBC

Typically, treatment progress for primary or metastatic tumors is made based on histology biopsy, which has several drawbacks, such as limited access to intratumoral heterogeneity, making it an impractical method for long-term disease surveillance. On the other hand, liquid biopsy is less invasive, simple to do, and doesn't require highly skilled medical workers. It can also be repeated frequently with minimum side effects [142]. Detecting CTC clusters in peripheral blood vessels in patients with MBC is a potential biomarker that strengthens the predictive value of counting single CTCs and evaluating treatment efficacy [133, 142]. About 3.4 percent of all CTCs are clustered, and 35 and 50 percent of patients with MBC have clusters. Also, clusters have a shorter half-life in the bloodstream (6–10 min compared to 25–30 min for single CTC) [143, 144].

Numerous studies have shown that CTC clusters have a greater potential for metastatic spread than single CTCs. Still, the degree of this disparity varies from 20 up to more than 100 times, and in mouse models, it was responsible for 50–97% of metastatic tumors [21, 145]. The capability of clusters to extravasate and survive in adverse environments, as well as their structural deformability, vascular shunts that allow them to circulate, their hybrid epithelial-mesenchymal phenotype, their stem cell characteristics, and the concentration on genes associated with replication and growth, and their improved cellular viability, all contribute to the explanation of the clusters' metastatic potential. The dimensions and density of groups in the blood affect all of those factors [22, 133]. Due to that, the detection of CTC clusters has been related to poor prognosis in MBC patients [37, 142]. Multiple studies showed that enumeration of CTC clusters has independent prognostic significance and increases predictive value to enumeration of CTC alone at baseline and follow-up [38, 133]. Table 2 compares the results of five studies that investigated the connection between CTC clusters and the prognosis of MBC patients. The presented Table 2 provides evidence that majority of investigations indicate a connection between the existence of CTC clusters and unfavorable results, including decreased PFS and OS, as well as an elevated hazard of disease progression and mortality. Moreover, mentioned studies in Table 2 reveals that CTC clusters exhibit a greater prevalence in specific BC subtypes, such as HER2-positive and triple-negative, and that the quantity and dimensions of these clusters may impact patient survival.

**Table 2:** Five Studies showed that CTC-cluster enumeration has independent prognostic significance and adds predictive value

Study Name	Study TYPE	Number of patients	Treatment GROUPS	Primary endpoint results	Breast cancer subtype	CTC cluster detection method	Statistical significance of results
Jansson et al. [36]	A prospective observational study (cohort study)	Fifty-two patients newly diagnosed with a first metastatic incident were scheduled to begin first-line therapy in the metastatic context	They did not have different treatment groups but followed the patients who received first-line therapy in the metastatic context according to their BC subtype (hormone receptor-positive, HER2-positive, or triple-negative)	The CTC clusters detected in the blood at 1–3 months and 6 months were linked to a shorter PFS. At 1–3 months, CTC clusters were also linked to shorter OS. Still, at 6 months, OS could not be assessed because every patient in the group that had clusters died before patients in the group without clusters	They followed the patients according to their BC subtype (hormone receptor-positive, HER2-positive, or triple-negative). They found that CTC clusters were less commonly detected at baseline and between 1 and 3 months in patients with hormone receptor-positive tumors than in HER2-positive and triple-negative BC patients. Still, at 6 months, there was no significant variance	Used CellSearch analysis to detect apoptotic CTC, CTC clusters, and WBC-CTCs. They defined CTC clusters as two or more CTCs close to each other within the same image field	The CTC clusters in the blood at 1–3 and 6 months were linked to shorter PFS. At 1–3 months, CTC clusters were also related to shorter OS. Patients with clusters had a greater risk of cancer progression and mortality compared to those who had no CTC clusters at any stage during the trial duration
Wang et al. [37]	Cohort study	Before beginning a new therapy, there were one hundred twenty-eight female patients with metastatic BC	They did not have different treatment groups but followed the research types and received various therapies according to their physician's discretion	Baseline CTC clusters significantly correlated with reduced PFS outcome. Additionally, the longitudinal analysis found that CTC clusters had more predictive value than CTC enumeration alone. Also, changes in both CTCs and CTC clusters from baseline to the first follow-up predicted patient survival	NA	Used the CellSearch System to detect CTCs and CTC clusters. They defined CTC clusters as two or more CTCs close to each other within the same image field	Baseline CTC clusters significantly correlated with reduced PFS outcome. CTC clusters had more excellent predictive value than CTC enumeration alone. Changes in both CTCs and CTC clusters significantly predicted patient survival. CTC cluster size significantly correlated with patient outcomes [115]
Larsson et al. [38]	A prospective observational study (cohort study)	One hundred fifty-six patients with recently diagnosed metastatic BC	They did not have different treatment groups but followed the patients who received systemic therapy according to their physician's discretion	Patients with CTC clusters at baseline had poorer OS and PFS rates than those without CTC clusters. The hazard ratio of both PFS and OS increased with the presence of CTC clusters during systemic therapy	NA	Used CellSearch technology to identify CTCs and CTC clusters. They defined CTC clusters as two or more CTCs close to each other within the same image field	The patients with CTC clusters at baseline had worse OS and PFS. PFS and OS hazard ratios increased when CTC clusters were present during therapy. Moreover, mortality was 11 times higher in patients with high CTC counts (CTCs 5) and CTC clusters compared to individuals without these characteristics
Costa et al. [133]	Cohort study	Fifty-four female metastatic BC patients	They did not have different treatment groups but followed the patients who received various therapies according to their physician's discretion and their BC subtype (HR + HER2-, HR + HER2 + , HR-HER2 + , or triple-negative)	At baseline, the CTC cluster showed a higher risk of disease progression and death. The PFS, OS, and survival time were also shorter. Furthermore, it was discovered that patients with a CTC cluster of four or more cells had a higher likelihood of disease progression than those with a CTC cluster of 2–3 cells (which, compared to patients without a CTC cluster, had a greater risk of disease progression)	They followed the patients according to their BC subtype (HR + HER2-, HR + HER2 + , HR-HER2 + , or triple-negative). They found that patients with the HR + HER2 subtype were more frequently found to have CTC clusters	Used the CellSearch System to isolate and count CTCs and CTC clusters. They defined CTC clusters as two or more CTCs close to each other within the same image field	At baseline, the CTC-cluster had a higher chance of disease progression and death, along with shorter PFS, OS, and survival time. Patients with a 4 + cell CTC-cluster had a greater probability of disease progression. The continuous existence of CTC clusters in the circulatory blood was related to shorter OS and a greater mortality risk
Paoletti et al. [134]	prospectively designed retrospective translational medicine study	They include 595 female patients with established BC and signs of metastatic cancer on clinical and/or radiographic assessments	They had two treatment groups: one that received eribulin mesylate (a chemotherapy drug) and one that received capecitabine (another chemotherapy drug)	They found that patients with doublets and clusters had statistically lower OS at baseline and the First Follow-up than those with couples only, clusters only, or no doublets or clusters. Also, CTC clusters were associated with a poorer prognosis, regardless of their detection time	NA	Used the CellSearch system to count the CTCs, classifying them as clusters when there are three or more and as doublets when there are two. They used the "revised" CellSearch CTC enumeration algorithm, which counts the number of CTCs in a couple or cluster and each CTC to determine the total CTC enumeration	Paoletti et al. found that patients with doublets and clusters had lower OS at baseline and the first follow-up. CTC clusters were linked to a worse prognosis, whether discovered at baseline or the first follow-up

Jansson et al. [36] looked at whether diagnostic information will be obtained from CTC clusters and apoptotic CTC in MBC together with CTC enumeration in all BC subtypes. According to time-dependent landmark analysis, patients who presented an increasing fraction of CTC clusters per CTC number in follow-up samples had a substantially worse prognosis. They concluded that independent of other predictive markers such as CTC numbers and BC subtype, Greater mortality was linked to the presence of apoptotic CTCs and CTC clusters at any given time. The study limitation was the small number of patients that hindered the statistical power and under presentation of HER2-positive and triple-negative subgroups despite being diagnosed more frequently with CTC clusters at baseline.

Wang et al. [37] The size of CTC-clusters and patient outcomes were found to be correlated in a time-dependent analysis using longitudinal CTC and CTC cluster records, in particular OS, and a higher risk of disease progression in relation to the size of the CTC cluster. This phenomenon was not observable in the PFS analysis but was present in OS analysis. They claimed that larger CTC clusters produced more metastatic foci. This result disproves the traditional consensus that the extremely tiny capillaries that separate the CTC clusters from the circulation are too thinto pass through, demonstrating that they can pass through capillaries by unfolding into single-cell chains [146]. The study limitations were (1) patient enrollment and treatment were not homogeneous because the clinical trial was not prospectively designed clinical trial. (2) Findings must be confirmed in larger, independent populations due to the small patient sample size and short follow-up. (3) Despite the lack of a standard for these tests, They proved that repeated CTC and CTC cluster measurements were stronger than single -point enumerations in predicting patient prognosis. Also, comprehensive assessments are required to determine how effectively these metrics work clinically.

Larsson et al. [40] identified CTCs and CTC clusters at baseline, 1, 3, and 6 months after starting systemic therapy. Patients with MBC who underwent a 6-month longitudinal analysis of dynamic changes of CTC and CTC cluster had better prognostication and therapy monitoring. They reported that CTC clusters detected patients with poorer outcomes and were independently and significantly predictive at all times. This study's positive advantage is the prospective design of a 6-month assessment for CTC clusters and serial CTC in women with recently diagnosed MBC and sampling before the initiation of first-line systemic treatment and systematic assessment at scheduled periods. The study limitation was the extended period of inclusion due to restrictive inclusion criteria, which contained exclusively recently diagnosed MBC patients prior to initiating first-line therapy, and the performance status score for the Eastern Cooperative Oncology Group ranges from zero to two.

Costa et al. [133] while at baseline, the existence of CTC cluster presented prognostic significance at follow-up, it didn't due to few patients from whom samples were obtained for follow-up (38 out of the 54 patients only 18.4% of them had CTC clusters) and the short time of some patients' follow-up. CTC cluster changes from baseline to follow-up could not forecast patient survival or progression. Their findings indicate that in patients with fewer than 20 CTCs, the existence of CTC clusters added prognostic information independent of CTC count. In contrast, in patients with more than 20 CTCs, CTC clusters had no predictive value. However, they suggested that a larger cohort study would be required to dress this issuedress this issue adequately.

Paoletti et al. [134] found that different disease locations and BC subtypes did not significantly differ in the frequency of CTC doublets and clusters. They concluded that neither clusters nor doublets significantly impact the course of first-line chemotherapy for patients with MBC and that the number of CTC present probably caused the poor correlation between clusters and OS. They must note that they used the "revised" CellSearch CTC enumeration algorithm. The number of CTCs in a doublet or cluster, as well as each individual CTC, were counted to determine the total CTC enumeration, so a patient who initially had only 1–4 CTCs (below the cutoff for positivity) might have more CTCs in the revised algorithm than classic algorithm also the power of their analysis is also limited by the size of the subgroup. They recommended that further predictive data for patients with MBC could be obtained through longitudinal evaluation of CTC doublets and clusters.

According to Jansson et al. [36] analysis of the prognostic importance of the existence of WBC-CTCs, at baseline or 1–3 months, patients with WBC-CTCs present did not significantly outlive patients without WBC-CTCs. In contrast, at 6 months, patients with WBC-CTCs had worse OS and PFS. According to Costa et al. [133], both at baseline and follow-up, WBC-CTCs were linked to a greater CTC count (more than 5 CTCs/7.5 mL); however, clustered WBC-CTC was unable to predict patient outcomes; this may be because of their small patient population and brief follow-up period. Also, it was noted that CD44 + CTC clusters were linked to a worse OS than CD44-CTC clusters [22, 143]. Additionally, Jansson et al. [36] discovered that the existence of apoptotic CTCs at 1–3 and 6 months following the start of treatment was linked to higher mortality regardless of other prognostic markers, including CTC counts and BC subtype. Paoletti et al. [107] showed no evidence of a predictive role for apoptotic CTCs either at baseline or in follow-up samples.

### Prevention of CTC cluster formation in BC patients

CTCs, are infrequent cellular entities within the blood's periphery that can form clusters in BC patients [3]. These clusters can lead to the spread of cancer and are, therefore, a target for prevention. A few research projects on mitigating metastasis. In an in vivo study using the 4T1 mouse model of BC metastasis, they found that injecting clinical thrombolytic agent urokinase-type plasminogen activator into the host animals was an effective way to prevent CTC cluster assembly and extend overall host survival by about 20% in comparison to control animals [147].

Moreover, the clinical significance of ICAM1 expression to patient outcomes was determined by analyzing two distinct cohorts of BC patients. High levels of ICAM1 mRNA expression in breast tumors correlated with poorer distant metastasis-free survival. ICAM1 promotes metastasis by triggering cellular pathways associated with stemness and cell cycle. Additionally, disrupting ICAM1 interactions significantly hinders the formation of CTC clusters and tumor cell trans-endothelial migration. Therefore, ICAM1 presents a potential therapeutic target for initiating TNBC metastases [148].

To limit the quantity or size of CTC clusters, a variety of commonly used chemotherapeutic drugs target the cytoskeleton microtubules and induce cell cycle arrest during mitosis. An in vitro study investigated the impact of mitotic arrest on the ability of BC cells to form clusters. It was discovered that the chemotherapy medicines vinorelbine and paclitaxel, which target microtubules, impair the ability of MCF-7 cancer cells to aggregate. They saw that MCF-7 BC cells aggregated poorly and formed loose clusters when experimentally synchronized and blocked in metaphase. Because microtubule-targeting anticancer medications prevent cancer cells from aggregating, they may lessen the chance that circulating tumor cells would metastatically spread [149].

Here are some drugs for BC (Table 3), including their mechanisms of action. These treatments include ADGRG1 inhibitors, estrogen receptor alpha inhibitors, chemotherapy drugs, customized drug screening, VEGF inhibitors, pro-angiogenic treatment, and other medications for metastasis. Each therapy has a unique mechanism of action that could prevent the formation of CTC clusters or improve the delivery of chemotherapy drugs and oxygen to tumors. Moreover, the methods by which various treatments operate differ; they can block the function of specific proteins involved in cell–cell adhesion or metastasis, eliminate cancer cells and halt their spread, improve blood flow to tumors, or obstruct the development of new blood vessels that supply oxygen and nutrients to malignancies. These therapies aim to either inhibit the formation of CTC clusters or enhance the delivery of chemotherapy drugs and oxygen to tumors. Further research is essential to determine each patient's most effective course of action.

**Table 3:** A therapeutic target to decrease breast cancer metastasis

Treatment	Description	Mechanism
ADGRG1 inhibitors [150]	ADGRG1 is a protein that actively facilitates the process of tumorigenesis, invasion/migration, and cell–cell adhesion in cells associated with triple-negative breast cancer	Inhibitors of ADGRG1 could prevent CTC cluster formation by blocking its role in cell–cell adhesion
Estrogen receptor alpha (ER/ESR1) inhibitors [151]	Mutations in ER/ESR1 have been detected in 20–40% of metastatic breast cancers resistant to endocrine therapy. These mutations are linked to unfavorable outcomes	Inhibitors of ER/ESR1 could prevent CTC cluster formation by blocking its role in metastasis
Chemotherapy drugs [152]	Chemotherapy is the prevailing approach for managing systemic therapy in triple-negative breast cancer (TNBC) patients	It can still effectively prevent CTC cluster formation by killing cancer cells and preventing their spread
Customized drug screening [153]	A culture assay of CTC derived from patients can be a valuable tool for evaluating anticancer drugs to guide therapy for personalized treatment	This approach allows for evaluating drug response using patient-derived CTC cultures obtained from a liquid biopsy
VEGF inhibitors [55]	The protein VEGF serves to activate the genesis of novel blood vessels	The growth of new blood vessels that provide tumors with oxygen and nutrients exists through inhibitors that target VEGF
Pro-angiogenic treatment [55]	The pro-angiogenic treatment promotes the genesis of novel blood vessels	This treatment could improve blood flow to tumors, allowing for better chemotherapy drugs and oxygen delivery
Epigen [154]	Clusters promoted the expression of the low-affinity EGFR ligand epigen, which promotes effective metastatic outgrowth and is exhibited in the highest levels in metastatic tumors	The metastatic outgrowth was reduced by 94% upon Epigen knockdown. Additionally, there was a decrease in the size of lung metastases, although the total number was not affected
Plakoglobin [155]	Plakoglobin, a component of desmosomes and adherence junctions, was overexpressed 219-fold more in CTC clusters and was associated with lower distant metastasis-free survival (p = 0.008)	Eliminating intercellular contacts crucial for cluster formation was achieved through Plakoglobin knockdown, and the count of tumor-derived CTC clusters decreased in experimental mouse tumors
Keratin-14 [45]	Desmosome and hemidesmosome adhesion complex genes, which control cell–matrix adhesion, cell–cell adhesion, and immune evasion, were abundant in keratin 14 cells	The mean number of metastases was seven times lower in keratin 14 knockdown tumors than in control tumors
ICAM-1 [156]	ICAM-1 is a transmembrane glycoprotein crucial for melanoma cells' adhesion to the endothelial monolayer [124]	ICAM-1 expression is increased with an increase in tumor cell adhesion. Thus, treatment with specific anti-ICAM-1 antibodies decreases this effect
Hemophilic CD44 [157]	CD44, a multifaceted transmembrane glycoprotein, facilitates the epithelial-mesenchymal transition process [126]	CD44-positive cells exhibited a heightened propensity for tumor formation in immunodeficient mice relative to their CD44-negative counterparts
Na + /K + ATPase inhibitor [158, 159]	Na + /K + ATPase exists on the cell membrane; its expression is highly expressed in breast cancer cases	Ouabain treatment inhibits the expression of Na + /K + ATPase in mice, which inhibits distant tumor formation in multiple mouse metastasis models

## Authors' conclusion

CTC clusters, while rare, play a significant role in the progression and spread of BC. These clusters' unique biological characteristics and molecular profiles increase their survival, invasion, and stemness abilities. Various techniques based on physical or immunological properties can be used to detect and isolate CTC clusters from the blood of BC patients. CTC clusters have been linked to poorer clinical outcomes, such as reduced survival rates and an increased likelihood of disease progression. As such, CTC clusters may be useful as biomarkers for monitoring treatment response, predicting prognosis, and guiding personalized therapy. However, many challenges still need to be solved in CTC clusters' study and clinical application, including their low frequency, heterogeneity, dynamicity, and standardization. Further research is required to better understand the origin, composition, mechanisms of action, and potential therapeutic targets of CTC clusters in BC and to develop more sensitive and reliable methods for their detection and characterization.

## Data Availability

No data associated with the manuscript.
